# Biophysical Correlates of Enhanced Immunogenicity of a Stabilized Variant of the Receptor Binding Domain of SARS-CoV-2

**DOI:** 10.1021/acs.jpcb.2c07262

**Published:** 2023-02-15

**Authors:** Kawkab Kanjo, Gopinath Chattopadhyay, Sameer Kumar Malladi, Randhir Singh, Sowrabha Jayatheertha, Raghavan Varadarajan

**Affiliations:** † Molecular Biophysics Unit (MBU), Indian Institute of Science, Bengaluru 560012, India; ‡ Mynvax Private Limited, Fourth Floor, Brigade MLR Center, 50, Vanivilas Rd, Gandhi Bazaar, Basavanagudi, Bangalore, Karnataka 560004, India

## Abstract

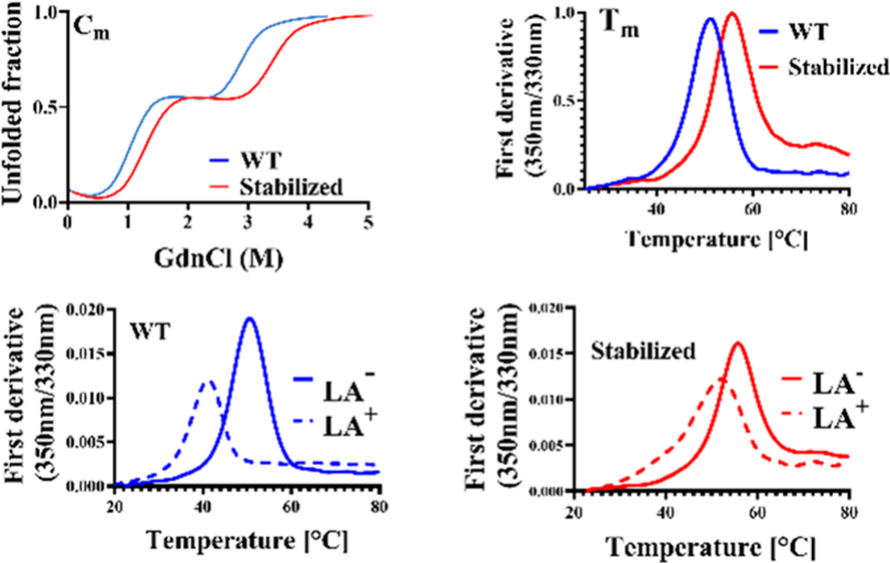

The receptor binding domain (RBD) of SARS-CoV-2 is the primary target of neutralizing antibodies. We have previously reported the design and characterization of a mammalian cell expressed RBD derivative, mRBD1-3.2, that has higher thermal stability and greatly enhanced immunogenicity relative to the wild type mRBD. The protein is highly thermotolerant and immunogenic and is being explored for use in room temperature stable Covid-19 vaccine formulations. In the current study, we have investigated the folding pathway of both WT and stabilized RBD. It was found that chemical denaturation of RBD proceeds through a stable equilibrium intermediate. Thermal and chemical denaturation is reversible, as assayed by binding to the receptor ACE2. Unusually, in its native state, RBD binds to the hydrophobic probe ANS, and enhanced ANS binding is observed for the equilibrium intermediate state. Further characterization of the folding of mRBD1-3.2, both in solution and after reconstitution of lyophilized protein stored for a month at 37 °C, revealed a higher stability represented by higher *C*_m_, faster refolding, slower unfolding, and enhanced resistance to proteolytic cleavage relative to WT. In contrast to WT RBD, the mutant showed decreased interaction with the hydrophobic moiety linoleic acid. Collectively, these data suggest that the enhanced immunogenicity results from reduced conformational fluctuations that likely enhance *in vivo* half-life as well as reduce the exposure of irrelevant non-neutralizing epitopes to the immune system.

## Introduction

Coronaviruses are the causative agent of several zoonotic outbreaks that have occurred since 2002. The recent ongoing pandemic caused by SARS-CoV-2 has, as of October 2022, resulted in ∼620 million confirmed cases, and 6.5 million deaths have been reported globally.^1^

SARS-CoV-2 is an enveloped RNA virus that uses its spike glycoprotein (S) to facilitate entry into the host cell. The spike protein is a homotrimeric class I fusion glycoprotein which is comprised of two subunits: S1, which contains the N-terminal domain (NTD) and the receptor binding domain (RBD), and S2, which contains the heptad repeats HR1 and HR2. The virus enters host cells through the binding of the viral RBD with the host cell surface receptor, angiotensin converting enzyme (ACE2). This process triggers conformational changes in the spike protein, leading to membrane fusion, mediated by the S2 subunit.^2^ Therefore, the RBD plays a critical role in virus attachment and infectivity. Further, several serological studies and clinical trials have shown that most of the potent neutralizing antibodies elicited through vaccination or natural infection are targeted toward the RBD of the spike glycoprotein.^3−7^

The present study probes the folding pathway of the SARS-CoV-2 RBD, using mammalian cell expressed RBD-based subunit vaccine candidates, which were previously shown to be more tolerant to thermal stresses than a stabilized spike ectodomain.^8^ The relevance of protein folding studies for biological function and molecular evolution is widely recognized. Despite its crucial role in vaccine design, little is known about the folding pathway of the RBD. Here, we report thermodynamic and kinetic characterization of the SARS-CoV-2 RBD, primarily using nanodifferential scanning fluorimetry (nanoDSF). Many proteins are known to populate intermediate states, which may aid folding through restricting the number of accessible conformations.^9,10^ In the current study using nanoDSF coupled with ANS binding studies, we found an equilibrium intermediate state, populated at intermediate denaturant concentrations, which is stable at 25 °C. An RBD variant, mRBD1-3.2, which was isolated and characterized in a previous study, exhibited higher thermal stability (Δ*T*_m_ = 7 °C), enhanced thermal tolerance (stable in adjuvanted formulations at 45 °C for over a week), and elicited sera with an over 100-fold increase in neutralizing antibody titers relative to the corresponding WT RBD.^11^ The sera neutralized diverse pseudoviruses as well as replicative virus from variants of concern including Alpha, Beta, Delta, and Omicron BA.1.^11,12^ Hamsters immunized with mRBD1-3.2 were protected from lethal challenge with a high dose of SARS-CoV-2 virus. Upon challenge, hamsters exhibited minimal weight loss and showed lower pathology scores compared to the control unimmunized animals.^11^

mRBD1-3.2 contains three mutations (A348P, Y365W, and P527L).^11^ To further probe the stabilizing effect of these mutations and rationalize the enhanced immunogenicity, we investigated the folding pathway and proteolytic sensitivity of mRBD1-3.2. We observed faster refolding rates and slower unfolding rates of the stabilized variant compared to the WT protein as well as higher resistance to chemical denaturation and tryptic digestion. We also showed that the stabilized RBD has reduced sensitivity to destabilization by the hydrophobic ligand, linoleic acid, which has previously been suggested to bind to the SARS-CoV-2 spike.^13^

## Materials and Methods

### Reagents

Ultrapure MB grade guanidium chloride (GdnCl) and hen egg white lysozyme (HEWL) were obtained from USB Corporation (Cleveland, OH). HEWL was diluted in 1 × PBS at pH 7.4 and used without any further purification. Linoleic acid was obtained from Sigma-Aldrich, cat no. L1012.

### mRBD Protein Expression and Purification

The mRBD1-WT sequence consists of residues 331–532 of the SARS-CoV-2 RBD fused to a C-terminal cleavable His tag, while the stabilized RBD (mRBD1-3.2) sequence is the same as that of mRBD1-WT, residues 331–532, with incorporation of three stabilizing mutations, A348P, Y365W, and P527L, identified by YSD.^11^ The purifications of mRBD1-WT and mRBD1-3.2 were carried out as described previously.^8,11^ Briefly, transfections were performed with 1 μg of plasmid per 1 mL of Expi293F cells, complexed with ExpiFectamine293 and transiently transfected into Expi293F cells, followed by the addition of Enhancer 1 and Enhancer 2 post 16 h of transfection, according to the manufacturer’s protocol. Five days post transfection, culture supernatant was collected and 2-fold diluted with 1 × PBS (pH 7.4) and bound to a Ni-NTA column pre-equilibrated with 1 × PBS (pH 7.4). Proteins were affinity purified using Ni-NTA affinity chromatography by mixing 2 mL of Ni Sepharose 6 Fast Flow (GE Healthcare) with the supernatant. The unbound fraction was removed, and the resin was washed with a 10-column volume wash of wash buffer (1 × PBS, 25 mM imidazole, pH 7.4). The bound proteins were eluted with 300 mM imidazole in 1 × PBS (pH 7.4). The eluted fractions were pooled and dialyzed thrice using a 3–5 kDa (MWCO) dialysis membrane (Spectrum Laboratories) against 1 × PBS, at pH 7.4. The eluted fractions were subjected to 15% Tricine SDS-PAGE, and the protein concentration was determined.

### Isothermal Denaturation

Equilibrium unfolding experiments of mRBD1-WT and mRBD1-3.2 were carried out by nanoDSF (Prometheus NT.48) as described previously.^14,15^ Briefly, the changes in the fluorescence ratio (*F*_350_/*F*_330_) were monitored, after overnight incubation of 10 μM of protein at 25 °C in 1 × PBS containing various concentrations of the denaturant, guanidium chloride (GdnCl), to determine the stability parameters for chemical denaturation. GdnCl concentrations were calculated from measurements of the refractive index using a refractometer. The data were analyzed using Sigmaplot for Windows scientific graphing software, and the plots were fitted to a three-state unfolding model (N_3_ → 3D). mRBD is a monomer in the native state, while ACE2-hFc is present in a dimeric form. The fraction unfolded for all mRBD mutants was calculated as described previously.^15^

### Refolding and Unfolding Kinetics

Refolding and unfolding kinetics for mRBD1-WT and mRBD1-3.2 (both in solution and the lyophilized form kept at 37 °C, for one month followed by reconstitution in 1 × PBS) were monitored by nanoDSF (*F*_350_/*F*_330_) using PR.Time Control software (Prometheus NT.48) at 25 °C as described previously.^14,15^ To measure the rates of refolding from U → N, protein in 1 × PBS, at pH 7.4, was denatured in 4 M GdnCl and subsequently diluted to final concentrations of 0.4, 0.5, 0.6, 0.8, and 1.0 M GdnCl. The changes in signal were monitored as a function of time. To measure the unfolding kinetics from N → U, protein in a native buffer (1 × PBS, pH 7.4) was diluted into the same buffer containing 7 M GdnCl to a final concentration of GdnCl varying from 3 to 4 M, and the changes in the fluorescence ratio (*F*_350_/*F*_330_) were monitored as a function of time. To measure the unfolding kinetics from N → I, protein in native buffer (1 × PBS, pH 7.4) was diluted into the same buffer containing 4 M GdnCl to a final concentration of GdnCl varying from 1.8 to 2.2 M, and the changes in the fluorescence ratio (*F*_350_/*F*_330_) were monitored as a function of time. Refolding and unfolding kinetic traces of fluorescence intensity in all cases, as a function of time for RBD, were normalized from 0 to 1 between native and denatured baselines as described previously.^14−17^ The data for the mRBD proteins were analyzed using Sigmaplot for Windows scientific graphing software, and data were fit to a three parameter equation for exponential decay for refolding (*y* = *a*_0_ + *a*_1_ exp(−*kf*_1_*x*)) and a five parameter exponential for unfolding (*y* = *A*_0_ + *A*1 exp(−*ku*_1_*x*) + *a*_2_ exp(−*ku*_2_*x*)), yielding slow and fast phase rate constants as described previously, where *x* is the time of refolding/unfolding.

### Thermal Stability Measurement by nanoDSF of the mRBD Proteins

nanoDSF (Prometheus NT.48) was also used to probe the thermal unfolding of the mRBD proteins. The assays were carried out with 10 μM of each protein, and the apparent thermal stability (*T*_m_) was determined by monitoring the changes in the fluorescence ratio (*F*_350_/*F*_330_) as a function of temperature as described previously.^14,15,18^ Samples were heated from 20 to 95 °C with a ramp rate of 1 °C/min. For mRBD proteins, refolding was carried out in 0.5, 0.6, 0.8, and 1.0 M GdnCl, and refolded protein was subjected to thermal denaturation with native protein in 0.5, 0.6, 0.8, and 1.0 M GdnCl as controls. To probe the activity of the refolded mRBD proteins, the refolded proteins along with native proteins in the same GdnCl concentrations were incubated with ACE2 and subjected to thermal denaturation.

### Unfolding and Refolding Fluorescence Studies

Experiments on equilibrium GdnCl induced unfolding were carried out using a FluoroMax-3 (Horiba Jobin Yvon) spectrofluorometer. Measurements were carried out with 5 μM of protein in 1 × PBS buffer, at pH 7.4 and 25 °C. The proteins were unfolded by incubating with different concentrations of GdnCl (3.0 to 4.0 M) for 4 h at 25 °C prior to the fluorescence measurements. Refolding was carried out with a fixed concentration of the protein (5 μM) in 1 × PBS buffer at pH 7.4 denatured in 3.0 M GdnCl and subsequently diluted to the final denaturant concentrations. The samples were excited at 280 nm, and the emission was measured between 300 and 450 nm. For each experiment, a cuvette containing only buffer and the appropriate GdnCl concentration were used to calculate blank values, which were then subtracted from the sample values.

### ANS Binding

To investigate protein surface hydrophobicity, ANS binding studies were carried out. The fluorescence signal of ANS increases and is blue-shifted upon its binding to hydrophobic pockets or patches.^19^ The samples were incubated overnight with different GdnCl concentrations in a range from 0.0 to 4.4 M, followed by the addition of ANS (1:10 protein to ANS molar ratio) and further incubated for 30 min. Samples incubated with ANS were then excited at 390 nm, and the emission was recorded from 400 to 600 nm. A native hen egg white lysozyme (HEWL) negative control in 1 × PBS at pH 7.4 was used. For all of the samples, fluorescence intensities were corrected by subtracting the blank containing the corresponding GdnCl concentration and ANS in 1 × PBS at pH 7.4. The protein was at a fixed concentration of 5 μM in all cases.

### Trypsin Digestion

The proteins were dialyzed in autoclaved Milli-Q water and reconstituted in 50 mM Tris at pH 7.5 with 1 mM CaCl_2_ and incubated with TPCK treated trypsin (protein: TPCK trypsin = 50:1) at 37 °C for different time points. The reaction was stopped by adding SDS dye and boiling the samples for 10 min at 95 °C followed by SDS-PAGE.

### Linoleic Acid Binding

Aliquots of 10 μM mRBD proteins were incubated with different concentrations of linoleic acid in 1 × PBS at pH 7.4 and subjected to thermal denaturation from 20 to 95 °C with a ramp rate of 1 °C/min using nanoDSF (Prometheus NT.48). The apparent thermal stability (*T*_m_) was determined by monitoring the changes in the fluorescence ratio (*F*_350_/*F*_330_) as a function of temperature.

## Results

### mRBD1 Unfolds Through an Equilibrium Intermediate

**Figure 1:**
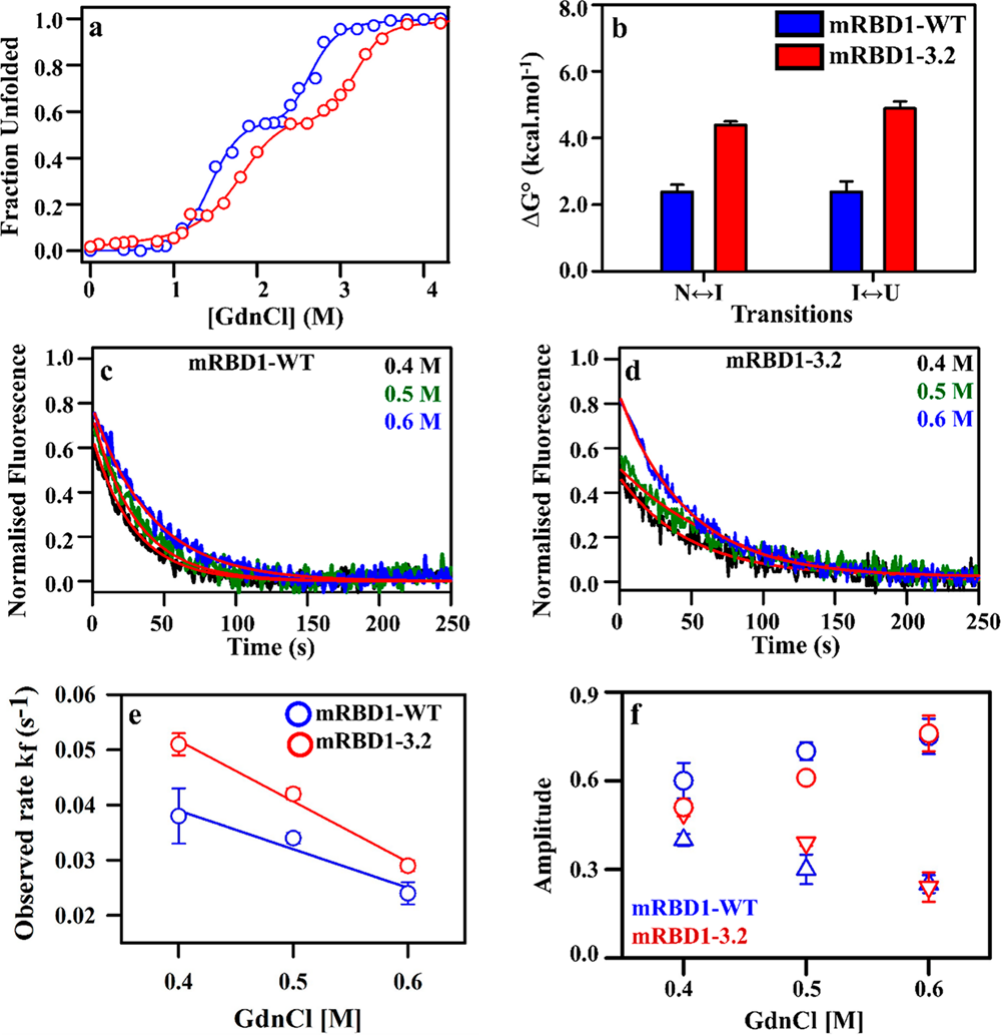
Chemical denaturation and refolding kinetics of wildtype (WT) and stabilized mRBD1. (a) Equilibrium denaturation profile of mRBD1-WT and stabilized mutant, mRBD1-3.2, with 10 μM protein in 1 × PBS, at pH 7.4 and 25 °C monitored using nanoDSF. The experimental data are shown in blue and red circles for mRBD1-WT and mRBD-1-3.2, respectively, while the fit is shown in blue and red lines for mRBD1-WT and mRBD-1-3.2, respectively. The theoretical curves were obtained by fitting all the melts with three-state unfolding models. (b) The estimated values of Δ*G*° for different transitions at 25 °C for mRBD1-WT (blue) and mRBD1-3.2 (red). (c,d) Refolding of mRBD1-WT and mRBD1-3.2 from U → N follows single exponential kinetics. Representative refolding kinetic traces of (c) mRBD1-WT and (d) mRBD1-3.2 at 10 μM protein concentration obtained at 0.4, 0.5, and 0.6 M final GdnCl concentrations are shown in black, green, and blue, respectively, while the fits are shown in red. (e) The dependence of the estimated refolding rate constants on denaturant concentration for the U → N transition for mRBD1-WT (blue circles) and mRBD1-3.2 (red circles). The estimated refolding rate constant and the refolding *m* values at zero denaturant concentration are determined to be 0.067 s^–1^ and −0.07 M^–1^ s^–1^, respectively, for mRBD1-WT and 0.096 s^–1^ and −0.11 M^–1^ s^–1^, respectively, for mRBD1-3.2. (f) The amplitudes A0 (triangles) and A1 (circles) of burst and fast phases as a function of GdnCl concentration. Data for mRBD1-WT and mRBD1-3.2 are shown in blue and red colors, respectively. The error bars, wherever shown, represent the standard deviation from two independent experiments.

The equilibrium unfolding experiment of mRBD1-WT and mRBD1-3.2 was carried out as explained in the Materials and Methods. The unfolded fraction of mRBD1-WT and mRBD1-3.2 in the presence of different concentrations of GdnCl was plotted as a function of denaturant concentration (Figure 1a). An equilibrium intermediate state was observed for mRBD1-WT. The experiment was performed in triplicate. The stabilized variant mRBD1-3.2 was 2.5 kcal/mol more stable than mRBD1-WT, for the intermediate to unfolded state (I → U) transition, and 2.0 kcal/mol more stable than the mRBD1-WT, for the native to intermediate state (N → I) transition (Figure 1b, Table S1). The midpoint of chemical denaturation (*C*_m_), Δ*G*^0^, and *m* values and are listed in Table S1. The equilibrium denaturation experiment was also performed in reverse, by unfolding then refolding the protein back to different final GdnCl concentrations ranging from 4.4 to 0.3 M, and the same profile, as in equilibrium unfolding *C*_m_, was obtained (Figure S1a,b).

### RBD Refolding and Unfolding Kinetics

Refolding kinetics of mRBD1-WT and mRBD1-3.2 were also monitored by time-course fluorescence spectroscopy at 25 °C using differential scanning fluorimetry (DSF). The refolding of mRBD1-WT and mRBD1-3.2 follow single exponential kinetics. Refolding reactions were carried out at three different GdnCl concentrations for mRBD1-WT and mRBD1-3.2 (Figure1, Table S2). The estimated refolding rate constants showed high dependence on GdnCl concentration (Figure 1e, Table S2). The estimated refolding rate constant and the refolding *m* values extrapolated to zero denaturant concentration are determined to be 0.067 s^–1^ and −0.07 M^–1^ s^–1^, respectively, for mRBD1-WT and 0.096 s^–1^ and −0.11 M^–1^ s^–1^, respectively, for mRBD1-3.2 (Table S2). With an increase in GdnCl concentration, there is an increase in the refolding phase amplitude at the expense of the burst phase (Figure 1f, Table S2). The unfolding from the native to the unfolded state of mRBD1-WT follows biphasic exponential kinetics (Figure S2). The unfolding was carried out at three different GdnCl concentrations, and representative kinetic traces obtained at 3.2, 3.4, and 3.6 M are shown in Figure S2a,c. Since unfolding was rapid under these conditions, the rate constants of unfolding of both mRBD1-WT and mRBD1-3.2 for N → U could not be accurately determined.

### Biphasic Unfolding Kinetics for mRBD1 Between Native and Intermediate States

**Figure 2:**
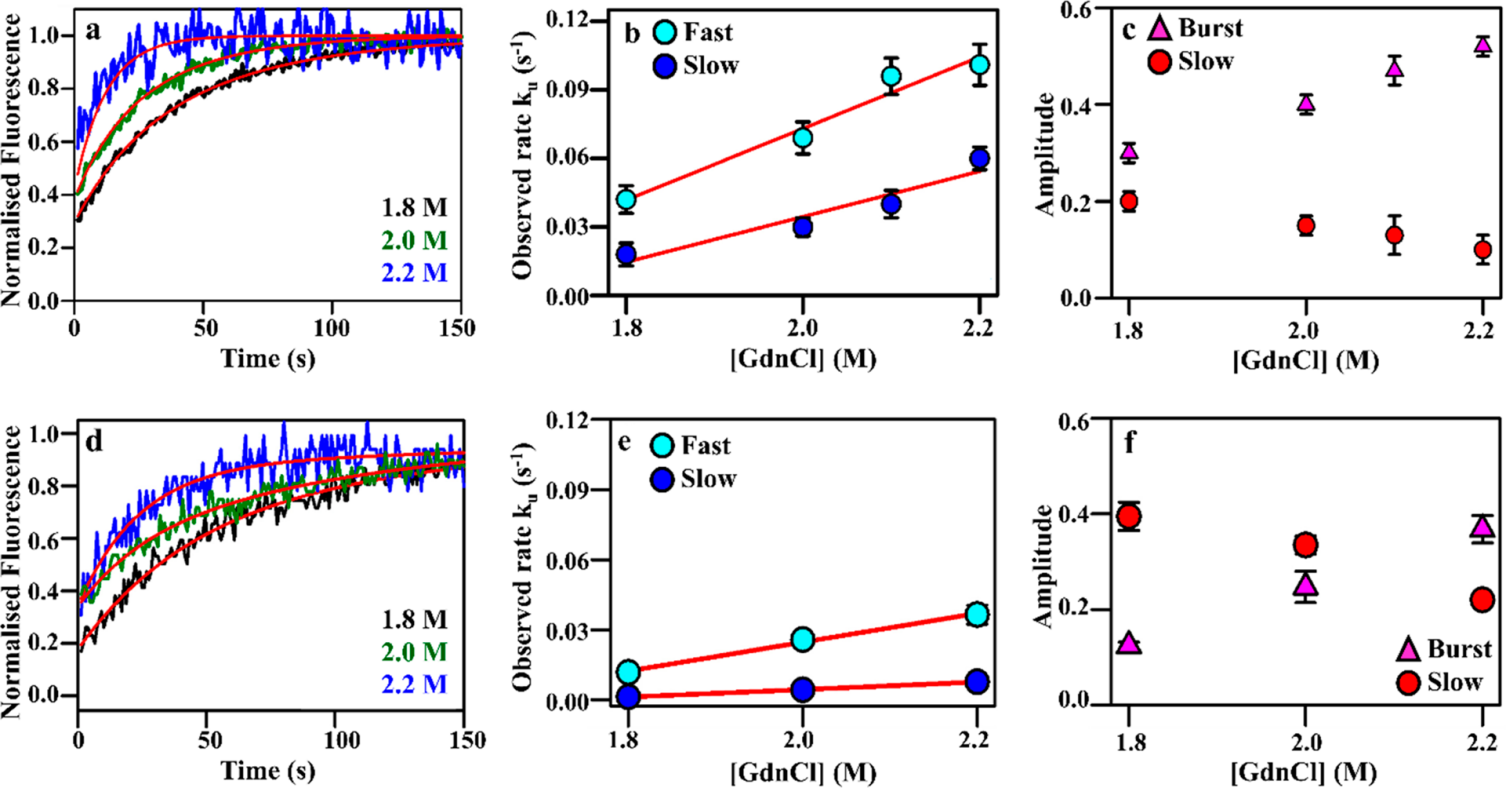
Unfolding kinetics of WT and stabilized mRBD1 (N → I). (a) Biphasic unfolding kinetics of mRBD1-WT from N→I, with a burst, fast, and a slow phase. Representative unfolding kinetic traces of mRBD1-WT at 10 μM protein concentration are shown. The experimental unfolding kinetic traces obtained at 1.8, 2.0, and 2.2 M final GdnCl concentration are shown in black, green, and blue, respectively, while the fits are shown in red. (b) The dependence of the estimated unfolding rate constants on denaturant concentration for the N→I transition. The estimated rate constant for mRBD1-WT of the fast phase at zero denaturant concentration is determined to be 0.06 s^–1^, while that of the slow phase is 0.008 s^–1^. The unfolding *m* values of the transition states of the fast and the slow phases were calculated to be 0.16 M^–1^ s^–1^ and 0.10 M^–1^ s^–1^, respectively. (c) The amplitudes of the burst (pink triangles) and slow (red circles) phases as a function of GdnCl concentration. (d) Biphasic unfolding kinetics of mRBD1-3.2 from N→I, with a burst, fast, and a slow phase. Representative unfolding kinetic traces of mRBD1-3.2 at 10 μM protein concentration are shown. The experimental unfolding kinetic traces obtained at 1.8, 2.0, and 2.2 M final GdnCl concentration are shown in black, green, and blue, respectively, while the fits are shown in red. (e) The dependence of the estimated unfolding rate constants for the N→I transition on the denaturant concentration. The estimated rate constant for mRBD1-3.2 of the fast phase at zero denaturant concentration is determined to be 0.012 s^–1^, while that of the slow phase is 0.003 s^–1^. The unfolding *m* values of the transition states of the fast and the slow phases were calculated to be 0.20 M^–1^ s^–1^ and 0.13 M^–1^ s^–1^, respectively. (f) The amplitudes of the burst (pink triangles) and slow (red circles) phases as a function of GdnCl concentration are shown. The error bars, wherever shown, represent the standard deviation from two independent experiments.

**Figure 3:**
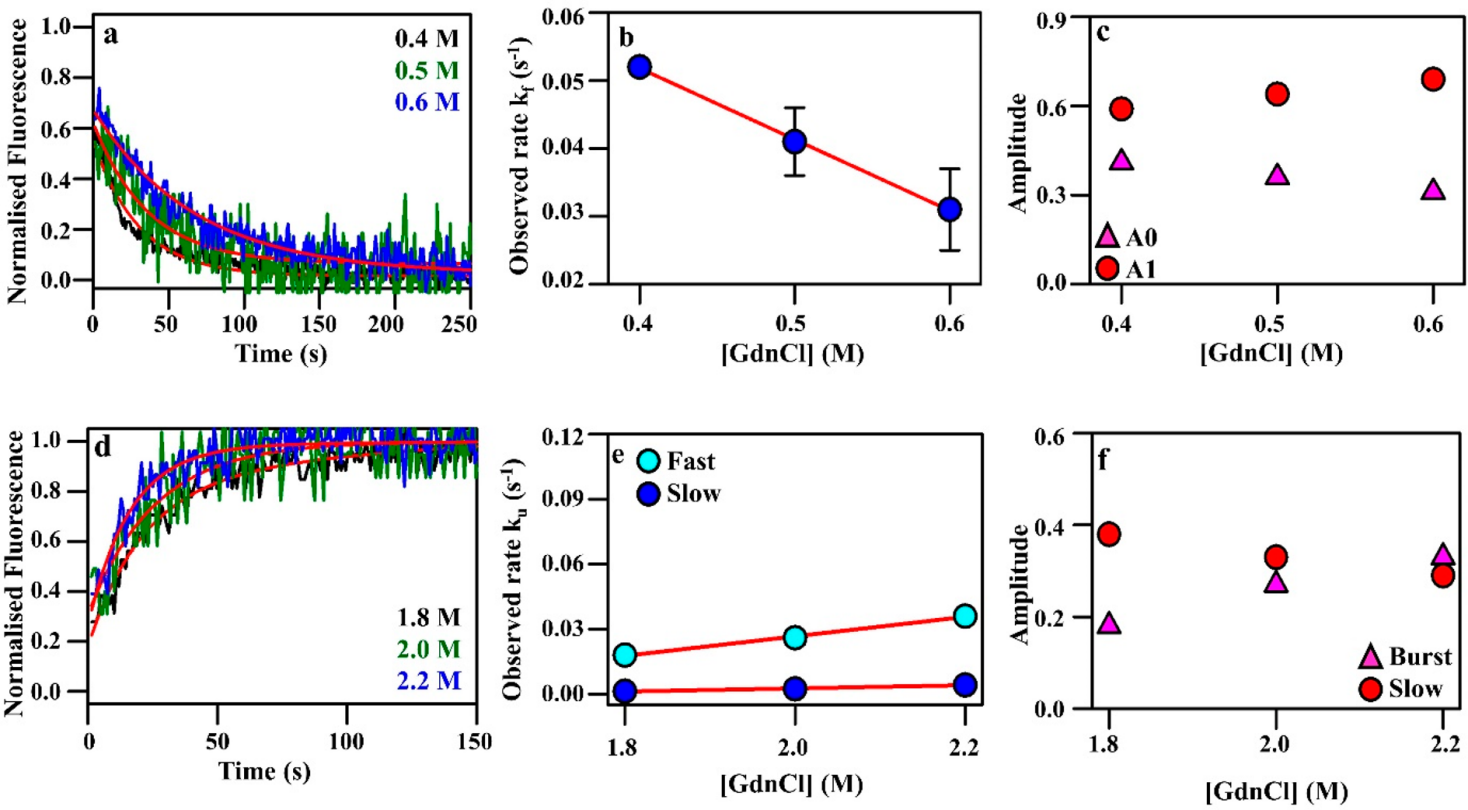
Refolding and unfolding kinetics following resolubilization of lyophilized, stabilized mRBD1-3.2 after storage at 37 °C for one month. (a) Refolding kinetics of mRBD1-3.2 for the U→N transition at 10 μM protein concentration. The experimental refolding kinetic traces obtained at 0.4, 0.5, and 0.6 M final GdnCl concentration are shown in black, green, and blue, respectively, while the fits are shown in red. (b) The dependence of the estimated refolding rate constants on denaturant concentration for the U→N transition. The estimated refolding rate constant and the refolding *m* values at zero denaturant concentration are determined to be 0.094 s^–1^ and −0.11 M^–1^ s^–1^, respectively. (c) The amplitudes of the A0 (pink triangles) and A1 (red circles) phases as a function of GdnCl concentration. (d) Biphasic unfolding kinetics of mRBD1-3.2 from N→I, with a burst, fast, and slow phase. Representative unfolding kinetic traces of mRBD1-3.2 at 10 μM protein concentration are shown. The experimental unfolding kinetic traces obtained at 1.8, 2.0, and 2.2 M final GdnCl concentrations are shown in black, green, and blue, respectively, while the fits are shown in red. (e) The dependence of the estimated unfolding rate constants for the N→I transition on denaturant concentration. The estimated unfolding rate constant for mRBD1-3.2 of the fast phase at zero denaturant concentration is determined to be 0.035 s^–1^, while that of the slow phase is 0.004 s^–1^. The unfolding *m* values of the transition states of the fast and the slow phases were calculated to be 0.10 M^–1^ s^–1^ and 0.07 M^–1^ s^–1^, respectively. (f) The amplitudes of the burst (pink triangles) and slow (red circles) phases with an increase in GdnCl concentration. The error bars, wherever shown, represent the standard deviation from two independent experiments.

The unfolding kinetics of mRBD1-WT and mRBD1-3.2 from N → I follow biphasic exponential kinetics, with a burst, fast, and a slow phase (Figure 2, Table S3), suggestive either of native state heterogeneity that might arise from multiple glycoforms or of parallel unfolding pathways. Unfolding reactions were carried out at four different GdnCl concentrations for mRBD1-WT and three different GdnCl concentrations for mRBD1-3.2. Representative unfolding kinetic traces of mRBD1-WT and mRBD1-3.2 obtained at 1.8, 2.0, and 2.2 M final GdnCl concentrations are shown in Figure 3a and d, respectively. The estimated unfolding rate constants and amplitudes were plotted as a function of GdnCl concentration. The unfolding of mRBD1-WT is fast, with an estimated rate constant of 0.06 s^–1^ for the fast phase at zero denaturant concentration, while that for the slow phase is 0.008 s^–1^ (Table S3). The unfolding *m* values of the transition states of the fast and the slow phases were calculated to be 0.16 M^–1^ s^–1^ and 0.10 M^–1^ s^–1^, respectively, for mRBD-1 WT (Table S3). The unfolding of mRBD1-3.2 is relatively slower, with an estimated rate constant of 0.012 s^–1^ for the fast phase at zero denaturant concentration, while that for the slow phase is 0.003 s^–1^ (Table S3). The unfolding *m* values of the transition states of the fast and the slow phases were calculated to be 0.20 M^–1^ s^–1^ and 0.13 M^–1^ s^–1^, respectively, for mRBD1-3.2 (Table S3). The amplitudes of the burst phase increase, while that of the slow phase decreases with an increase in GdnCl concentration, as shown in Figure 3c for mRBD1-WT and Figure 3f for mRBD1-3.2 (Table S3).

### mRBD1-3.2 Folding Kinetics is Unchanged Even after Storage for One Month in the Lyophilized State at 37 °C

mRBD1-3.2 protein was dialyzed against 1 × PBS, lyophilized, and then incubated for one month at 37 °C. After sample reconstitution in water, unfolding and refolding kinetics were carried out to compare the integrity of 37 °C stored lyophilized protein with that of freshly prepared, unlyophilized protein. The refolding of lyophilized mRBD1-3.2 protein that had been stored at 37 °C was found to follow single exponential kinetics. All of the refolding reactions were carried out at three different GdnCl concentrations, of 0.4, 0.5, and 0.6 M. Representative refolding kinetic traces of mRBD1-3.2 at 37 °C (lyophilized and incubated for 1 month at 37 °C prior to reconstitution) at 10 μM protein concentration are shown in Figure 3a. The estimated refolding rate constants and amplitudes were plotted as a function of GdnCl concentration (Figure 3b,c, Table S4). The estimated refolding rate constant and the refolding *m* values at zero denaturant concentration for mRBD1-3.2 at 37 °C for the U→N transition are determined to be 0.094 s^–1^ and −0.11 M^–1^ s^–1^, respectively. Increasing GdnCl concentration led to an increase in the slow phase amplitude and decrease in the burst phase amplitude (Figure 3c, Table S4).

The unfolding kinetics of mRBD1-3.2 at 37 °C from N→I follow biphasic exponential kinetics, with a burst, fast, and slow phase (Figure 3, Table S4). Three different GdnCl concentrations were used for the unfolding experiment of lyophilized and reconstituted mRBD1-3.2 (Figure 3d). The estimated unfolding rate constants and amplitudes from N→I were also plotted as a function of GdnCl concentration. The estimated rate constant for mRBD1-3.2 at 37 °C for the fast phase at zero denaturant concentration was found to be 0.035 s^–1^, while that of the slow phase was found to be 0.004 s^–1^ (Table S4). The unfolding *m* values of the transition states of the fast and the slow phases were calculated to be 0.10 M^–1^ s^–1^ and 0.07 M^–1^ s^–1^, respectively (Table S4). The amplitudes of the burst phase increase, while that of the slow phase decreases with an increase in GdnCl concentration, as shown in Figure 3f and Table S4.

### Refolded mRBD Proteins Bind ACE2 *in Vitro*

**Figure 4:**
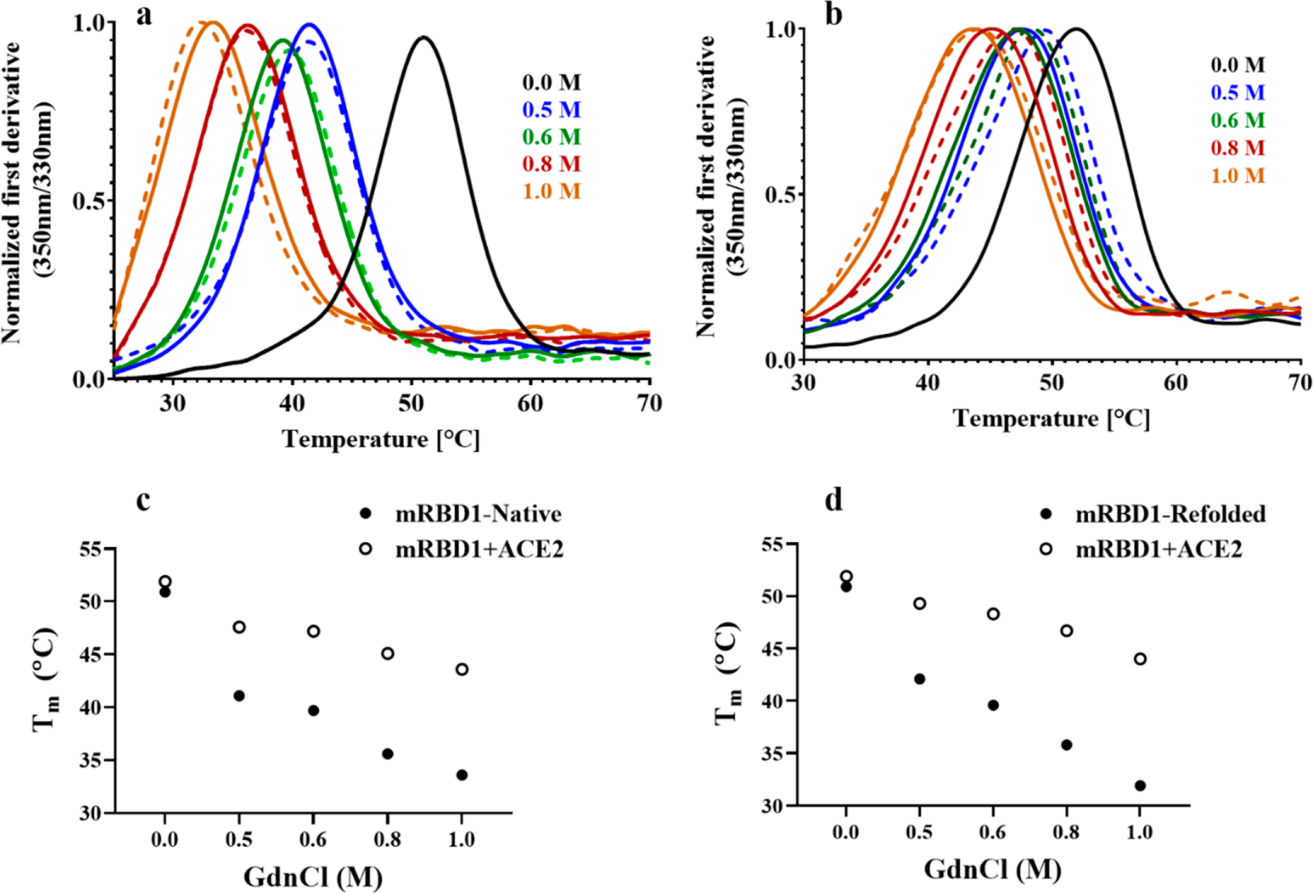
Thermal stability and ACE2 binding of native and refolded mRBD1-WT protein. (a) Thermal unfolding traces of 10 μM of refolded mRBD1-WT protein in 0.5 M, 0.6 M, 0.8 M, and 1.0 M GdnCl (dashed lines) and native mRBD1-WT protein in the same concentrations of GdnCl (solid lines). (b) Thermal unfolding in the presence of ACE2 of 10 μM of native mRBD1-WT protein in 0.5 M, 0.6 M, 0.8 M, and 1.0 M GdnCl (dashed lines) or refolded mRBD1-WT in the same concentrations of GdnCl (solid lines). In all cases, refolded and native proteins show a similar shift in the *T*_m_ in the presence of ACE2. Thermal unfolding of native protein in the absence of GdnCl (solid black lines). (c and d) Comparison of *T*_m_ values for native and refolded proteins in the absence or presence of ACE2, respectively.

**Figure 5:**
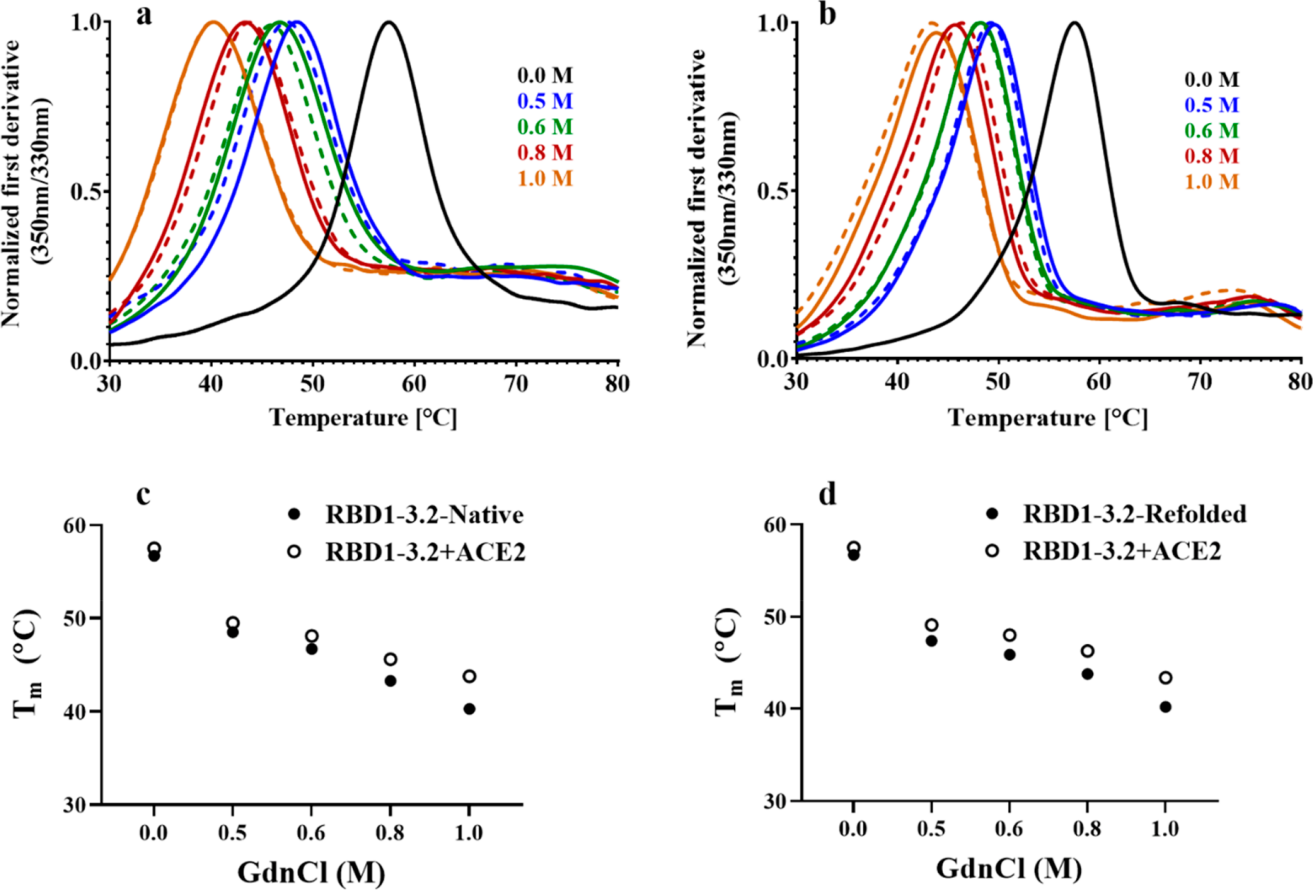
Thermal stability and ACE2 binding of native and refolded stabilized mRBD1 protein. (a) Thermal unfolding traces of 10 μM of refolded mRBD1-3.2 protein in 0.5 M, 0.6 M, 0.8 M, and 1.0 M GdnCl (dashed lines) and native mRBD1-3.2 protein in the same concentrations of GdnCl (solid lines). (b) Thermal unfolding in the presence of ACE2 of 10 μM of native mRBD1-3.2 protein in 0.5 M, 0.6 M, 0.8 M, and 1.0 M GdnCl (dashed lines) or refolded mRBD1-3.2 in the same concentrations of GdnCl (solid lines). In all cases, refolded and native proteins show a similar shift in the *T*_m_ in the presence of ACE2. Thermal unfolding of native protein in the absence of GdnCl (solid black lines). (c and d) Comparison of absolute *T*_m_ values for native and refolded proteins in the absence or presence of ACE2, respectively.

The refolded and the native mRBD1 proteins in the presence of different concentrations of GdnCl were also subjected to thermal denaturation, and the apparent *T*_m_ was calculated for mRBD1-WT (Figure 4a) and for stabilized RBD (Figure 5a). mRBD1 proteins showed clear thermal transitions even at 1.0 M GdnCl, confirming that they were in a folded conformation in the presence of GdnCl. Further, the ability of the refolded mRBD proteins and the native proteins in the presence of different concentrations of GdnCl to bind ACE2 was also qualitatively assessed using nanoDSF for mRBD1-WT (Figure 4b–d) and for mRBD1-3.2 (Figure 5b–d). In all cases, both the refolded and native mRBD1 proteins in the presence of GdnCl were able to bind to ACE2, indicating that the functional integrity of the proteins is still maintained after refolding.

### Characterization of the Native, Intermediate, and Unfolded States of mRBD1 Using Fluorescence Spectroscopy

**Figure 6:**
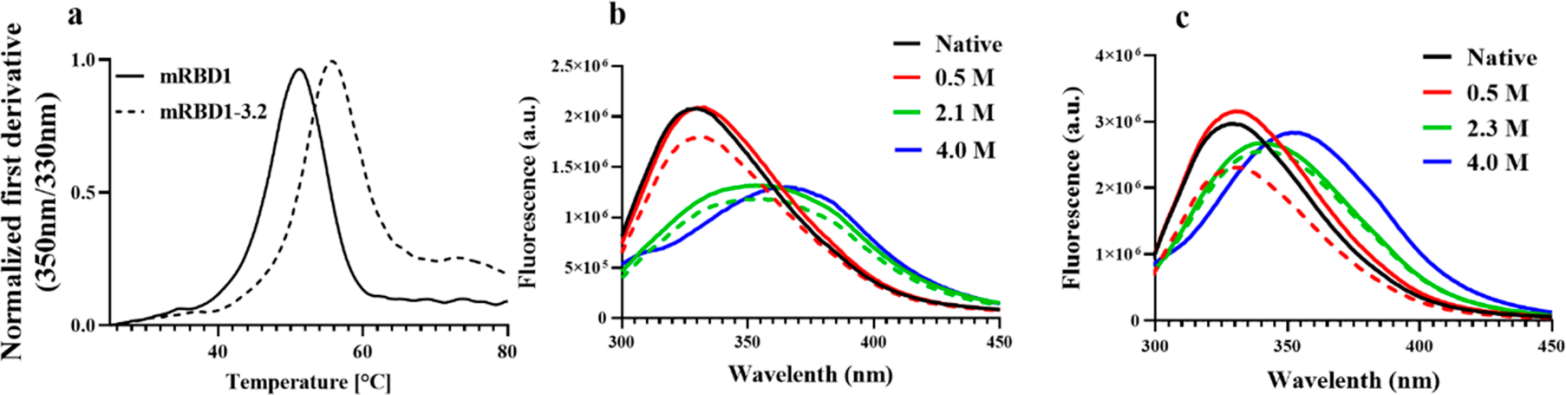
Thermal and chemical denaturation of WT and stabilized mRBD1, probed by fluorescence spectroscopy. (a) *T*_m_ of WT and mRBD1-3.2 measured by nanoDSF. Fluorescence profiles of mRBD1-WT (b) and mRBD1-3.2 (c) with 5 μM protein in 1 × PBS at pH 7.4 at 25 °C. Solid and dashed lines represent native and refolded mRBD1 protein incubated at various GdnCl concentrations. The samples were unfolded for 4 h in 4.0 M GdnCl before refolding. Refolding was initiated by diluting the samples with 1 × PBS to 0.5 M GdnCl. For accessing the intermediate state, samples were unfolded for 4 h before refolding by diluting the samples with 1 × PBS to a 2.1 M GdnCl concentration for mRBD1-WT and to a 2.3 M GdnCl concentration for mRBD1-3.2. The unfolded state fluorescence profile of mRBD1 at a GdnCl concentration of 4.0 M is shown as a blue solid line.

We compared the *T*_m_ of both mRBD1 and its stabilized version using nanoDSF. mRBD1-3.2 exhibited a higher *T*_m_ than WT by ∼7 °C (Figure 6a). The refolded mRBD1 proteins and the native proteins in 1.0 M GdnCl, display a similar fluorescence profile to that of the native protein in the absence of any GdnCl (Figure 6b for mRBD1-WT and Figure 6c for mRBD1-3.2). Increasing GdnCl concentration to 2.1 and 2.3 M where the intermediate state is populated for mRBD1-WT and mRBD1-3.2, respectively, resulted in a shift of the fluorescence peak to 350 nm. Upon complete unfolding of the mRBD1-WT protein in GdnCl concentrations ranging from 3.0 to 4.0 M, there is a further shift of the peak maximum to about 380 nm.

### mRBD1-3.2 Exhibits Higher Stability and Resistance to Proteolytic Cleavage than mRBD1-WT and Both Proteins Exhibit Binding to ANS in the Native State

**Figure 7:**
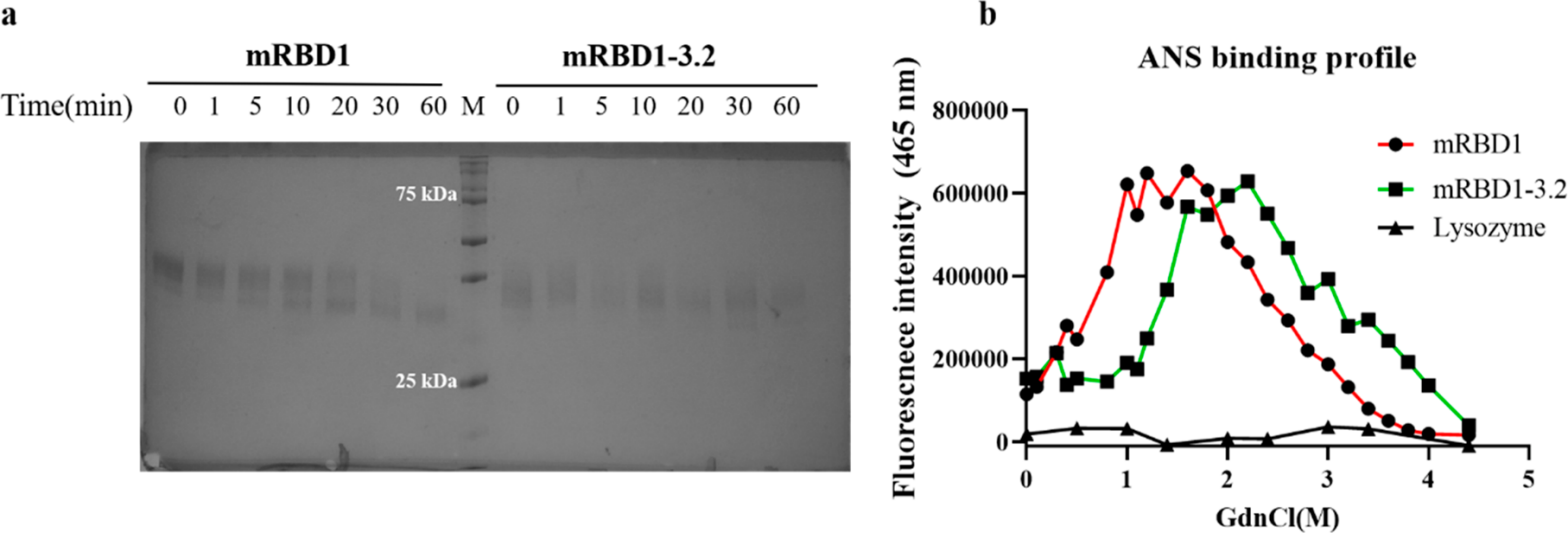
Proteolytic resistance and ANS binding of WT and stabilized mRBD1. (a) mRBD1 and mRBD1-3.2 proteins were incubated with trypsin at a ratio of 50:1 protein to trypsin at 37 °C for different time points and subjected to SDS-PAGE. (b) ANS fluorescence intensity at 465 nm following incubation with 5 μM of mRBD1 proteins at GdnCl concentrations ranging from 0.0 to 4.4 M as indicated above. The samples were incubated with GdnCl overnight at 25 °C before the addition of ANS at a final concentration of 50 μM and further incubation for 30 min. The samples were then excited at 390 nm, and the emission was monitored from 300 to 600 nm. Each point represents the fluorescence intensity at 465 nm of ANS bound to mRBD1 at a specific GdnCl concentration.

The three introduced mutations in mRBD1-3.2 render it more proteolysis-resistant than mRBD1. SDS-PAGE shows that mRBD1-3.2 protein is stable even after 1 h of incubation with trypsin at 37 °C (Figure 7a) in contrast to mRBD1, which is significantly degraded in 20 min.

We next investigated the ability of mRBD1-WT and mRBD1-3.2 proteins to bind to ANS, a hydrophobic, fluorescent molecular probe. ANS is used to probe the surface hydrophobicity of proteins. Hen egg white lysozyme (HEWL) was used as a control because, like most folded proteins, it does not exhibit binding to ANS in its native state. Relative to lysozyme, both mRBD1 proteins showed significant binding to ANS in their native state, suggesting that both proteins have exposed hydrophobic patches even in the absence of any denaturant (Figure 7b). Interestingly, ANS bound to mRBD1, relative to mRBD1-3.2, showed a larger blue shift, suggesting that the dye is bound in a more hydrophobic environment in WT mRBD1. With increasing GdnCl concentration, ANS binding increases until it displays a maximum fluorescence signal when the intermediate state is well populated (Figure 7b). Increasing the denaturant concentration further leads to a decrease in ANS binding until the protein is completely unfolded.

### mRBD1-3.2 Shows Reduced Binding to Destabilizing Fatty Acid, Linoleic Acid, Compared to WT

**Figure 8:**
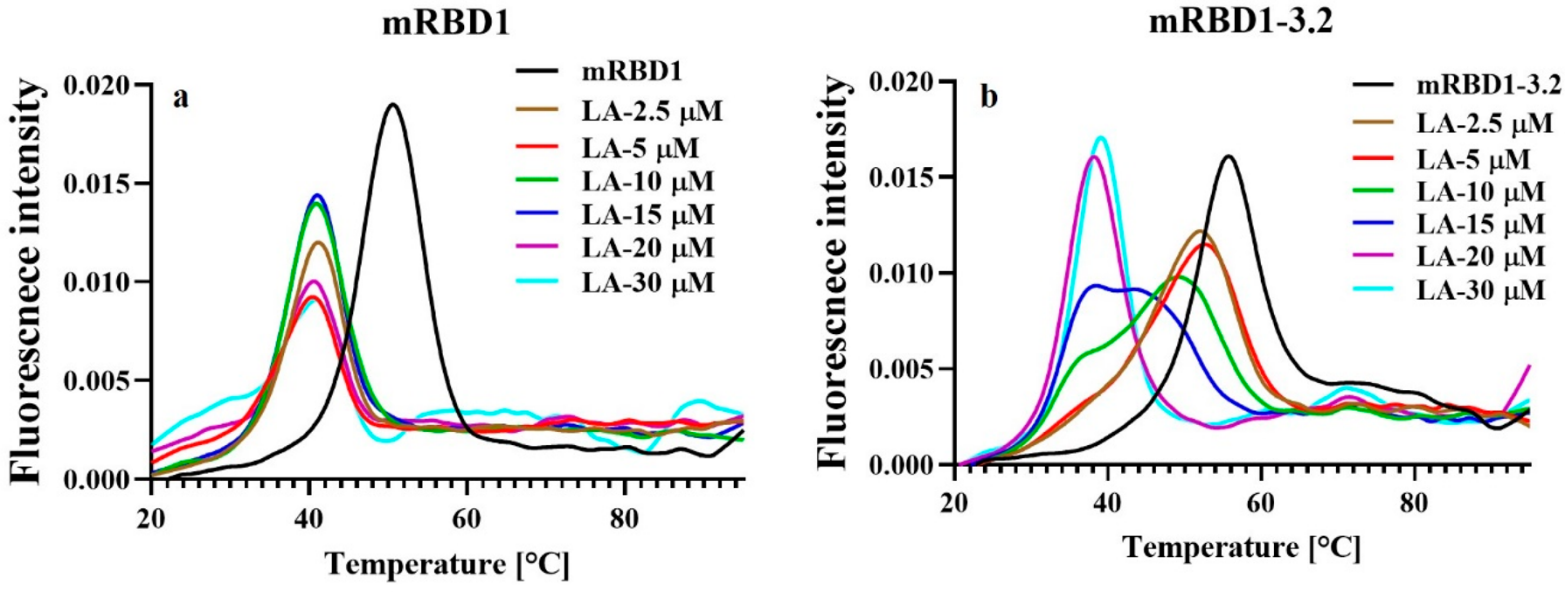
Interaction of WT and stabilized mRBD1 with linoleic acid assayed by nanodifferential scanning fluorimetry (nanoDSF). Thermal denaturation profiles of (a) mRBD1-WT and (b) mRBD1-3.2 protein without and with different concentrations of linoleic acid (LA), measured by nanoDSF.

It has previously been suggested that linoleic acid (LA) binds to a hydrophobic pocket located in the RBD of the SARS-CoV-2 spike.^13^ We therefore examined the binding of linoleic acid to both WT and stabilized RBD by measuring the thermal stability in the presence and absence of linoleic acid, with the expectation that the RBD thermal stability would be enhanced in the presence of LA. Surprisingly (Figure 8), the thermal stability decreased in the presence of LA, suggesting that LA binds preferentially to denatured or partially unfolded RBD relative to the native state. WT mRBD1 exhibited a large decrease in *T*_m_ at all concentrations of LA (Figure 8a). mRBD1-3.2 exhibited a small amount of destabilization in the presence of LA in a manner that depended on the LA concentration (Figure 8b). This difference in binding of LA between the two protein variants is likely due to the mutation Y365W present in mRBD1-3.2 proximal to the hydrophobic binding pocket of LA, which might obstruct LA binding^20^ as well as enhanced global stability, resulting in decreased conformational fluctuations, thus inhibiting LA binding.

## Discussion

It is clear that SARS-CoV-2 and its variants are firmly entrenched in humans as well as multiple other animal hosts. While several efficacious Covid-19 vaccines exist, they all require low temperature or ultra-low temperature storage, which are barriers to deployment in many parts of the world. There is a continued need for efficacious vaccines that do not require a cold chain, and this has led to isolation of stabilized forms of the spike protein and its constituent RBD domain.

Yeast surface display (YSD) coupled to second-site saturation suppressor mutagenesis (SSSM) in which incorporation of a single destabilizing mutation into each member of a single-site saturation mutagenesis library, followed by screening for suppressors, allows for robust and accurate identification of stabilizing mutations.^23,24^ This method was used to identify three stabilizing mutations (A348P, Y365W, and P527L) in the RBD that was displayed on the yeast surface. These mutations significantly improved RBD expression, thermal stability, thermal tolerance, and immunogenicity.^11^

In the present study, we have compared the unfolding and refolding kinetics, chemical denaturation, and LA binding of the mRBD1-WT with that of the stabilized mRBD1-3.2. The goal of these studies was to obtain insights into the reasons for enhanced immunogenicity of the stabilized RBD, and its ability to withstand prolonged exposure to high temperatures in both a lyophilized state as well as in solution.

**Figure 9:**
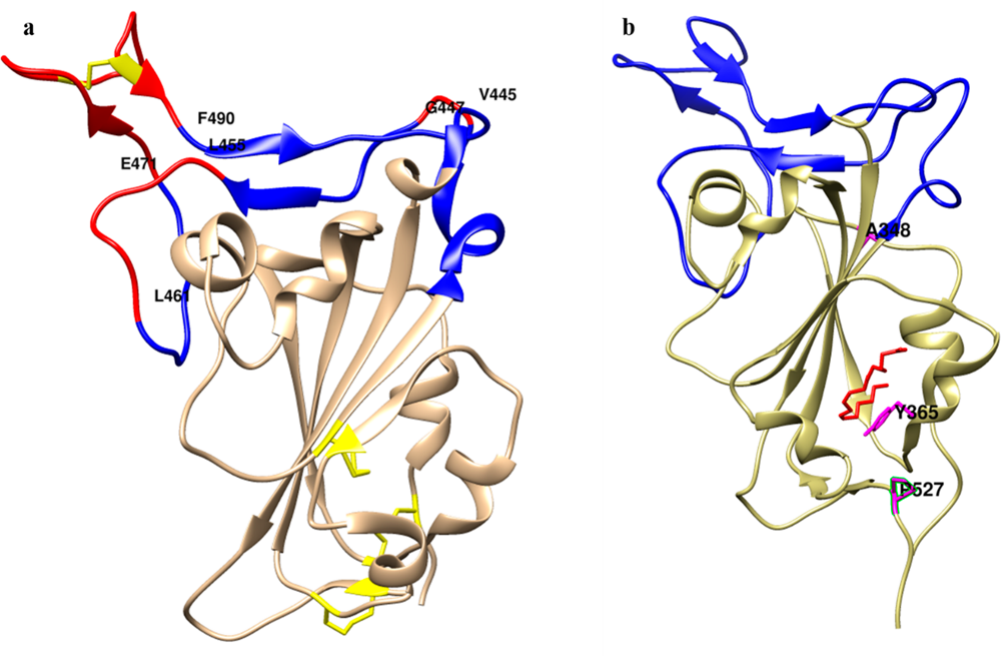
The structure of SARS-CoV-2 RBD bound to ACE2 and linoleic acid (a) RBD bound to ACE2 with the RBM regions that are disordered in the unbound structure but become ordered upon ACE2 binding highlighted in red, ordered RBM region shown in blue, disulfides shown in yellow sticks. Coordinates for bound and unbound RBD from the RBD:ACE2 crystal structure 6M0J^21^ and the unliganded spike Cryo-EM structure 6VYB,^22^ respectively. (b) Linoleic acid bound SARS-CoV-2 RBD. Linoleic acid (LA) moiety is indicated in red sticks and accommodated in a hydrophobic pocket gated by residue Y365, which is shown in magenta color. Y365, along with A348 and P527, are the three residues that are mutated in the stabilized mRBD1-3.2 and are highlighted in magenta with residue name and number. Coordinates taken from Cryo-EM structure of LA bound spike PDB ID: 6ZB4.^13^

Faster refolding and slower unfolding rates were observed for the stabilized RBD compared to mRBD1-WT. Upon lyophilization and incubation at 37 °C for one month, following resolubilization, mRBD1-3.2 exhibited similar refolding and unfolding kinetics to fresh protein in solution, consistent with its unchanged ACE2 binding observed previously.^11^ The equilibrium GdnCl denaturation profile of stabilized RBD showed an unusual three-state profile with higher stability than WT, represented by shifts in the position of both the N → I and I → U transitions to higher GdnCl concentration than that of mRBD1-WT. A shift was also observed in the ANS binding fluorescence profile, which interestingly showed that both WT and stabilized RBD show a high ANS binding fluorescence signal in their native states, compared to that of the lysozyme control. This suggests the presence of exposed hydrophobic patches on the RBD surface in its native state, which contains several loops. The positions of regions that are disordered in the absence of ACE2 but become ordered upon ACE2 binding are highlighted in Figure 9a. These differences are localized to an important region of RBD called the receptor binding motif (RBM), which consists of residues 438–505. The RBM, as the name suggests, is the region that contacts the ACE2 receptor. The majority of neutralizing antibodies also contact the RBM. The dynamic stability of mRBD1-3.2 was also probed by examining its resistance to proteolytic digestion by TPCK trypsin. The protein was resistant to digestion at 37 °C even after 60 min of incubation, in contrast to WT RBD, which showed significant degradation after 20 min. These stability results *in vitro* suggest that the stabilized protein may have a longer half-life *in vivo*, contributing to enhanced immunogenicity. mRBD1 harbors four pairs of disulfide bonds, of which three are in the core of RBD, thereby stabilizing the protein.^21^ In its native state, mRBD1 binds to ANS, a well-known probe for hydrophobic surfaces, and this binding reaches a maximum at the equilibrium intermediate state. The intermediate state did not bind to ACE2 at 2.0 and 2.2 M GdnCl as assessed by ELISA and nanoDSF, possibly because of the partial unfolding of ACE2 at these denaturant concentrations (Figure S3). The stabilized variant mRBD1-3.2 was 2.5 kcal/mol more stable than mRBD1-WT, for the intermediate to unfolded state (I → U) transition and 2.0 kcal/mol more stable than the mRBD1-WT, for the native to intermediate state (N → I) transition (Figure 1b, Table S1). At the present time, we do not have detailed structural information on the equilibrium intermediate. However, examination of the structure of the native, folded protein reveals that RBD has five twisted antiparallel beta strands connected via loops and short helices to form a well packed core, while the RBM is comprised of extended helices and loops which form the contact surface with ACE2. We hypothesize that the RBM unfolds first at low concentrations of the denaturant to reach a stable intermediate state that retains the tightly packed core, which is resistant to unfolding under denaturant concentrations less than 3.0 M. The locations of three stabilizing mutations A348P, Y365W, and P527L are highlighted in the structure of RBD shown in Figure 9. The A348P substitution is found in multiple other sarbecovirus RBDs including SARS-CoV-1. It likely stabilizes the protein through decreasing the conformational entropy of the unfolded state, thereby increasing the unfolded state free energy.^25^ The Y365W mutation partially fills in a cavity in the RBD structure, while the origin of the stabilizing effect of the P527L substitution is currently unclear, as this residue is disordered in the crystal structure of RBD bound to ACE2 (PDB ID 6m0j).^21^ None of these residues is part of the RBM. However, if RBM unfolding is coupled to structural destabilization of the core, it might explain why the native to intermediate transition is shifted to higher denaturant concentration for the stabilized protein. Destabilization of the unfolded state by the A348P substitution would also explain why the intermediate to unfolded state transition is shifted to higher denaturant concentration for the stabilized triple mutant RBD. Y365W was also identified as a stabilizing mutation in another study,^20^ which examined mutations previously identified by deep mutational scanning^26^ in conjunction with computational modeling. In the stabilized RBD, the lower red shift upon ANS binding (Figure 7b), resistance to proteolysis (Figure 7a), increase in *C*_m_ of the equilibrium intermediate, and reduced destabilization by linoleic acid (Figure 8a) all suggest that the stabilized RBD shows lower dynamic flexibility than WT RBD. *In vivo*, this probably results in increased half-life as well as lower exposure of irrelevant non-neutralizing epitopes, thus resulting in improved elicitation of neutralizing antibodies. The relationship between protein stability, protein dynamic fluctuations, and immunogenicity is poorly understood, and there are very few studies that have explored this systematically. Although we do not have definitive explanations for the increased immunogenicity of the stabilized RBD relative to WT, we have identified some biophysical correlates. In the future, we will attempt to image both WT and stabilized immunogens within B-cell follicles *in vivo*(27) to better understand the origins of the enhanced immunogenicity.

### Statistical Analysis

All the experiments are carried out in biological replicates (*n* = 3), and the listed errors are the standard errors derived from the values obtained for individual replicates. For the nanoDSF measurements, each experiment has been carried out thrice (*n* = 3), and the listed errors are the standard errors derived from the values obtained for individual replicates.

## Data Availability

The data relevant to the figures in the paper have been made available within the article and in the Supporting Information section. All unique/stable reagents generated in this study are available from the Lead Contact Raghavan Varadarajan (varadar@iisc.ac.in).

## Supporting Information Available

Thermodynamic parameters (*C*_m_, Δ*G*^0^, *m*) and kinetic parameters for unfolding and refolding of mRBD proteins; unfolding kinetics for mRBD-WT from the native to unfolded state and equilibrium denaturation profile of ACE2-Fc (PDF)
