# Supplementary material for: Biophysical Correlates of Enhanced Immunogenicity of a Stabilized Variant of the Receptor Binding Domain of SARS-CoV-2
Source: J Phys Chem B. 2023 Feb 15;127(8):1704–14. doi: 10.1021/acs.jpcb.2c07262 (PMC9942533; doi:10.1021/acs.jpcb.2c07262)
Supplement: Supplementary file 1 — jp2c07262_si_001.pdf [file jp2c07262_si_001.pdf]

# Supporting Information for Publication

# Biophysical Correlates of Enhanced Immunogenicity of a Stabilized Variant of the Receptor Binding Domain of SARS-CoV-2

Kawkab Kanjo[1], Gopinath Chattopadhyay[1], Sameer Kumar Malladi[1], Randhir Singh[2], Sowrabha Jayatheertha[2], Raghavan Varadarajan*[, 1,2]

[1] Molecular Biophysics Unit (MBU), Indian Institute of Science, Bengaluru-560012, India

[2] Mynvax Private Limited, Fourth Floor, Brigade MLR Center, 50, Vanivilas Rd, Gandhi Bazaar, Basavanagudi, Bangalore, Karnataka 560004, India

Correspondence:
Prof. Raghavan Varadarajan
Molecular Biophysics Unit,
Indian Institute of Science, Bangalore
Karnataka, India -560012
Email: varadar@iisc.ac.in
Phone: +91-80-22932612, FAX: +91-80-23600535

# Supplementary tables

**Table S1.** Thermodynamic parameters ($C_m$, $\Delta G^0$, $m$)[1] of mRBD proteins, determined by nanoDSF.

| Protein | Temperature (°C) | $C_{mN\text{-}I}$ (M) | $C_{mI\text{-}U}$ (M) | $\Delta G_{N\text{-}I}^0$ (kcal.mol$^{-1}$) | $\Delta G_{I\text{-}U}^0$ (kcal.mol$^{-1}$) | $m_{N\text{-}I}$ (kcal.mol$^{-1}$M$^{-1}$) | $m_{I\text{-}U}$ (kcal.mol$^{-1}$M$^{-1}$) |
|---|---|---|---|---|---|---|---|
| **mRBD1-WT** | 25 | 1.3±0.2 | 2.6±0.2 | 2.4±0.2 | 2.4±0.3 | 1.3±0.2 | 1.2±0.2 |
| **mRBD1-3.2** | 25 | 1.6±0.1 | 3.2±0.3 | 4.4±0.1 | 4.9±0.2 | 1.2±0.2 | 1.3±0.1 |

[1]Reported standard errors are derived from three independent experiments, each performed in triplicates.

**Table S2.** Kinetic Parameters for refolding from U→N of mRBD proteins at different GdnCl concentrations in 1xPBS, pH 7.4 and at 25 °C[1].

| Protein | GdnCl [M] | Refolding | | |
|---|---|---|---|---|
| | | a0 | a1 | $kf_1(s^{-1})$ |
| **mRBD1-WT[2]** | 0.4 | 0.40±0.02 | 0.60±0.06 | 0.038±0.005 |
| **mRBD1-WT** | 0.5 | 0.30±0.05 | 0.70±0.03 | 0.034±0.001 |
| **mRBD1-WT** | 0.6 | 0.25±0.03 | 0.75±0.06 | 0.024±0.002 |
| **mRBD1-WT** | 0.8 | 0.20±0.001 | 0.80±0.02 | 0.027±0.005 |
| **mRBD1-WT** | 1.0 | 0.15±0.002 | 0.85±0.01 | 0.020±0.0005 |
| **mRBD1-3.2[3]** | 0.4 | 0.49±0.01 | 0.51±0.01 | 0.051±0.002 |
| **mRBD1-3.2** | 0.5 | 0.39±0.01 | 0.61±0.01 | 0.042±0.001 |
| **mRBD1-3.2** | 0.6 | 0.24±0.05 | 0.76±0.06 | 0.029±0.001 |

[1]Reported standard errors are derived from three independent experiments, each performed in triplicates.

[2] The estimated refolding rate constant and the refolding *m*-value at zero denaturant concentration from U→N are determined to be 0.067 $s^{-1}$ and −0.07 $M^{-1}$ $s^{-1}$ for mRBD1-WT.

[3]The estimated refolding rate constant and the refolding *m*-value at zero denaturant concentration for mRBD1-3.2_37 °C from U→N are determined to be 0.094 $s^{-1}$ and −0.11 $M^{-1}$ $s^{-1}$ respectively.

**Table S3.** Kinetic Parameters for unfolding from N→I of mRBD1-WT and mRBD1-3.2 proteins at various GdnCl concentrations in 1xPBS, pH 7.4 and at 25 °C[1].

| GdnCl [M] | Unfolding (mRBD1-WT)[2] | | | | | Unfolding (mRBD1-3.2)[3] | | | | |
|---|---|---|---|---|---|---|---|---|---|---|
| | Fast Phase | | | Slow Phase | | Fast Phase | | | Slow Phase | |
| | A0 | A1 | $ku_1(s^{-1})$ | A2 | $ku_2(s^{-1})$ | A0 | A1 | $ku_1(s^{-1})$ | A2 | $ku_2(s^{-1})$ |
| **1.8** | 0.30± 0.02 | 0.50± 0.008 | 0.042 ±0.006 | 0.20± 0.02 | 0.018± 0.005 | 0.12± 0.01 | 0.48± 0.04 | 0.011 ±0.001 | 0.40± 0.04 | 0.0013± 0.0003 |
| **2.0** | 0.40± 0.02 | 0.45± 0.003 | 0.069 ±0.007 | 0.15± 0.02 | 0.030± 0.004 | 0.25± 0.03 | 0.43± 0.02 | 0.026 ±0.002 | 0.32± 0.02 | 0.0043± 0.0003 |
| **2.2** | 0.52± 0.02 | 0.38± 0.003 | 0.101 ±0.009 | 0.10± 0.03 | 0.060± 0.005 | 0.37± 0.03 | 0.41± 0.03 | 0.037 ±0.003 | 0.22± 0.02 | 0.0077± 0.0006 |

[1]Reported standard errors are derived from three independent experiments, each performed in triplicates.

[2] The unfolding rate constant of mRBD1-WT was estimated to be 0.06 $s^{-1}$ for the fast phase at zero denaturant concentration, while that for the slow phase is 0.008 $s^{-1}$. The unfolding *m*-values of the transition states of the fast and the slow phases were calculated to be 0.16 $M^{-1}$ $s^{-1}$ and 0.10 $M^{-1}$ $s^{-1}$, respectively.

[3] The unfolding rate constant of mRBD1-3.2 was estimated to be 0.012 $s^{-1}$ for the fast phase at zero denaturant concentration, while that for the slow phase is 0.003 $s^{-1}$. The unfolding *m*-values of the transition states of the fast and the slow phases were calculated to be 0.20 $M^{-1}$ $s^{-1}$ and 0.13 $M^{-1}$ $s^{-1}$, respectively.

**Table S4.** Kinetic Parameters for refolding from U→N and unfolding from N→I for lyophilized mRBD1-3.2 protein, stored for 1 month at 37 °C and then resolubilized in 1xPBS, pH 7.4 containing various concentrations of GdnCl at 25 °C[1].

| GdnCl [M] | Refolding[2] | | | GdnCl [M] | Unfolding[3] | | | | |
|---|---|---|---|---|---|---|---|---|---|
| | | | | | Fast Phase | | | Slow Phase | |
| | a0 | a1 | $kf_1(s^{-1})$ | | A0 | A1 | $ku_1(s^{-1})$ | A2 | $ku_2(s^{-1})$ |
| **0.4** | 0.41± 0.002 | 0.59± 0.004 | 0.052 ±0.001 | **1.8** | 0.18± 0.004 | 0.44± 0.006 | 0.018 ±0.002 | 0.38± 0.001 | 0.0013± 0.0003 |
| **0.5** | 0.36± 0.007 | 0.64± 0.008 | 0.041 ±0.005 | **2.0** | 0.27± 0.003 | 0.40± 0.002 | 0.026 ±0.003 | 0.33± 0.002 | 0.0024± 0.0002 |
| **0.6** | 0.31± 0.002 | 0.69± 0.001 | 0.031 ±0.006 | **2.2** | 0.33± 0.003 | 0.38± 0.004 | 0.036 ±0.002 | 0.29± 0.004 | 0.0042± 0.0002 |

[1]Reported standard errors are derived from three independent experiments, each performed in triplicates.

[2]The estimated refolding rate constant and the refolding *m*-value at zero denaturant concentration for mRBD1-3.2_37 °C from U→N are determined to be 0.094 $s^{-1}$ and −0.11 $M^{-1}$ $s^{-1}$ respectively.

[3] The estimated rate constant for mRBD1-3.2 of the fast phase at zero denaturant concentration is determined to be 0.035 $s^{-1}$, while that of the slow phase is 0.004 $s^{-1}$. The refolding *m*-values of the transition states of the fast and the slow phases were calculated to be 0.10 $M^{-1}$ $s^{-1}$ and 0.07 $M^{-1}$ $s^{-1}$, respectively.

## Supplementary Figures

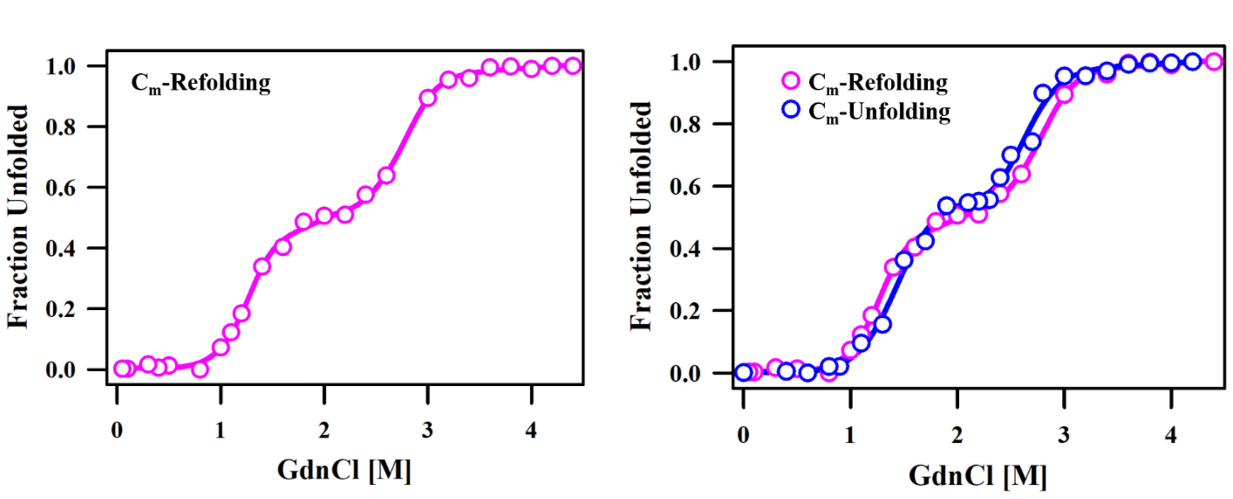


**Figure S1. Chemical denaturation profile of mRBD1-WT (N→U) and (U→N).** (a) Equilibrium denaturation profile of mRBD1-WT performed by unfolding the protein and then refolding it back to different denaturant concentrations, at pH 7.4, 25 °C and monitored using nanoDSF. The experimental data are shown in pink circle while the fit is shown in pink line. The theoretical curves were obtained by fitting all the melts with three-state unfolding models. (b) Superposition of equilibrium denaturation profiles of mRBD1-WT obtained by unfolding and refolding. The experimental data are shown in blue and pink circles for unfolding and refolding $C_m$, respectively, while the fit is shown in blue and pink lines for unfolding and refolding $C_m$, respectively. The refolding data for WT in part b is identical to that shown in part a and is repeated to facilitate comparison with the unfolding trace.

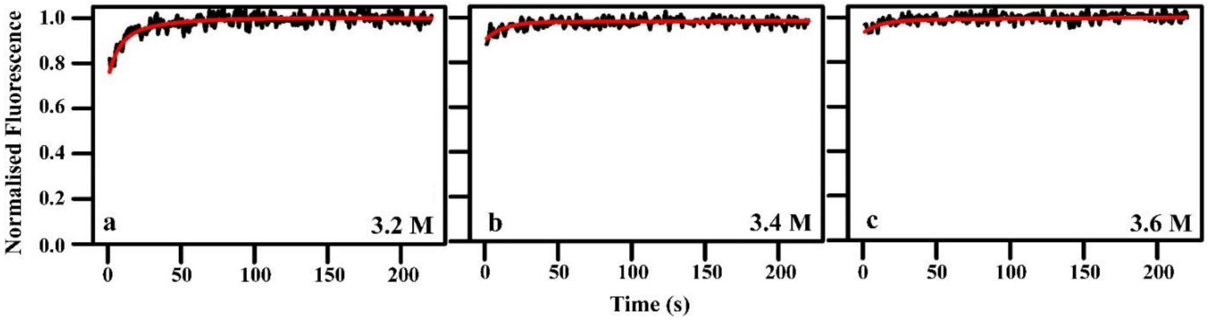


**Figure S2. Unfolding kinetics of mRBD1-WT (N→U).** (a-c) Unfolding of mRBD1-WT from N→U follows biphasic exponential kinetics with a large burst phase that precluded accurate determination of unfolding rate constants. Representative unfolding kinetic traces of mRBD1-WT at 10 µM protein concentration with 3.2 M (a), 3.4 M (b), and 3.6 M (c) final GdnCl concentration are shown in black, while the fits are shown in red.

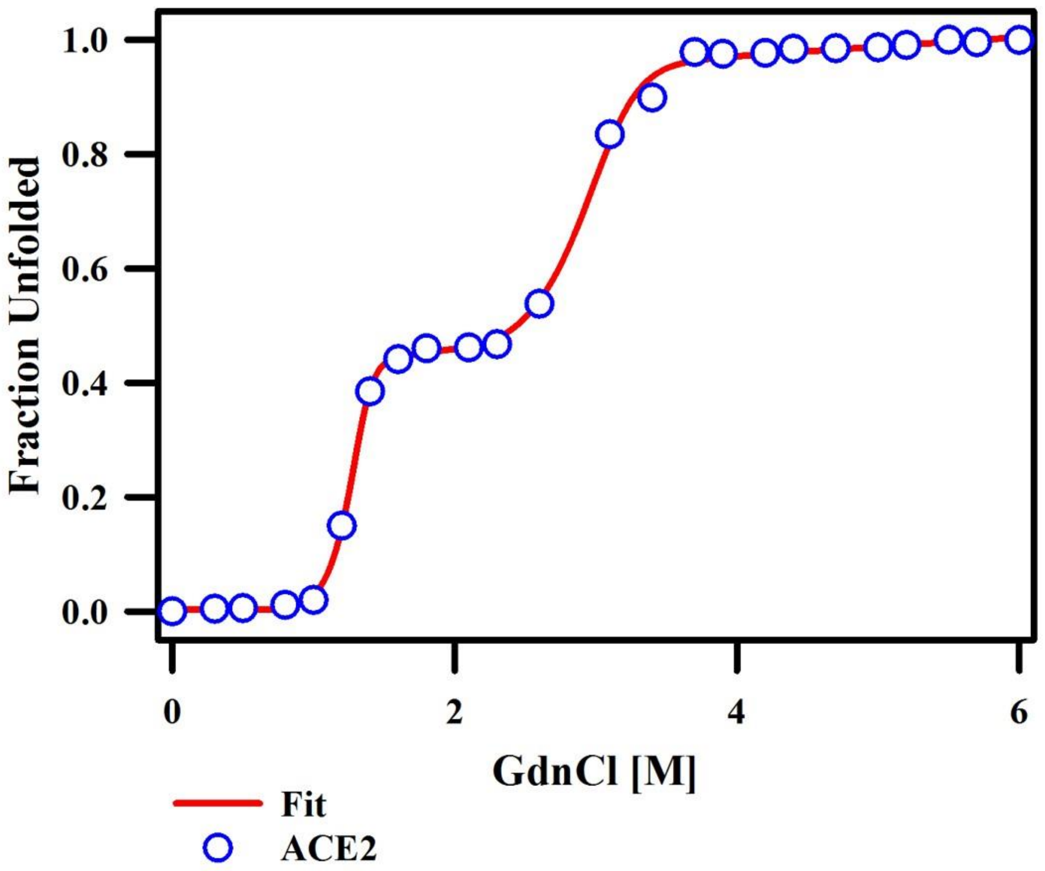


**Figure S3. Equilibrium denaturation profile of ACE2-Fc.** Equilibrium denaturation profile of ACE2-Fc with 10 µM protein in 1xPBS, pH 7.4, at 25 °C using nanoDSF. The experimental data are shown in blue circles, while the fit is shown in a red line. The theoretical curves were obtained by fitting all the melts with three-state unfolding models.
